# Suboptimal but intact integration of Bayesian components during perceptual decision-making in autism

**DOI:** 10.1186/s13229-025-00639-3

**Published:** 2025-01-13

**Authors:** Laurina Fazioli, Bat-Sheva Hadad, Rachel N. Denison, Amit Yashar

**Affiliations:** 1 Department of Special Education, University of Haifa, Haifa, Israel; 2 Department of Psychological and Brain Sciences, Boston University, Boston, USA

**Keywords:** Autism spectrum disorder, Decision-making, Bayesian perception, Suboptimality

## Abstract

**Background:**

Alterations in sensory perception, a core phenotype of autism, are attributed to imbalanced integration of sensory information and prior knowledge during perceptual statistical (Bayesian) inference. This hypothesis has gained momentum in recent years, partly because it can be implemented both at the computational level, as in Bayesian perception, and at the level of canonical neural microcircuitry, as in predictive coding. However, empirical investigations have yielded conflicting results with evidence remaining limited. Critically, previous studies did not assess the independent contributions of priors and sensory uncertainty to the inference.

**Method:**

We addressed this gap by quantitatively assessing both the independent and interdependent contributions of priors and sensory uncertainty to perceptual decision-making in autistic and non-autistic individuals (*N* = 126) during an orientation categorization task.

**Results:**

Contrary to common views, autistic individuals integrated the two Bayesian components into their decision behavior, and did so indistinguishably from non-autistic individuals. Both groups adjusted their decision criteria in a suboptimal manner.

**Limitations:**

This study focuses on explicit priors in a perceptual categorization task and high-functioning adults. Thus, although the findings provide strong evidence against a general and basic alteration in prior integration in autism, they cannot rule out more specific cases of reduced prior effect – such as due to implicit prior learning, particular level of decision making (e.g., social), and level of functioning of the autistic person.

**Conclusions:**

These results reveal intact inference for autistic individuals during perceptual decision-making, challenging the notion that Bayesian computations are fundamentally altered in autism.

**Supplementary Information:**

The online version contains supplementary material available at 10.1186/s13229-025-00639-3.

## Main text

In acknowledgment of the ongoing discourse regarding terminology about individuals diagnosed with autism, we used “autistic individuals” and “non-autistic individuals” in line with recent conventions.

## Background

Autism Spectrum Disorder is a group of neurodevelopmental disorders with an unknown etiology. Although autism encompasses a wide range of symptoms, it is primarily characterized by atypical social cognitive capacities, such as theory of mind and cognitive empathy [1]. Recently, there has been growing interest in sensory processing in autism as a core phenotype [2, 3]. Despite evidence demonstrating sensory symptoms and perceptual alterations in autistic people [4, 5], whether and how a single mechanism can explain the various symptoms of autism remains unknown.

**Fig. 1:**
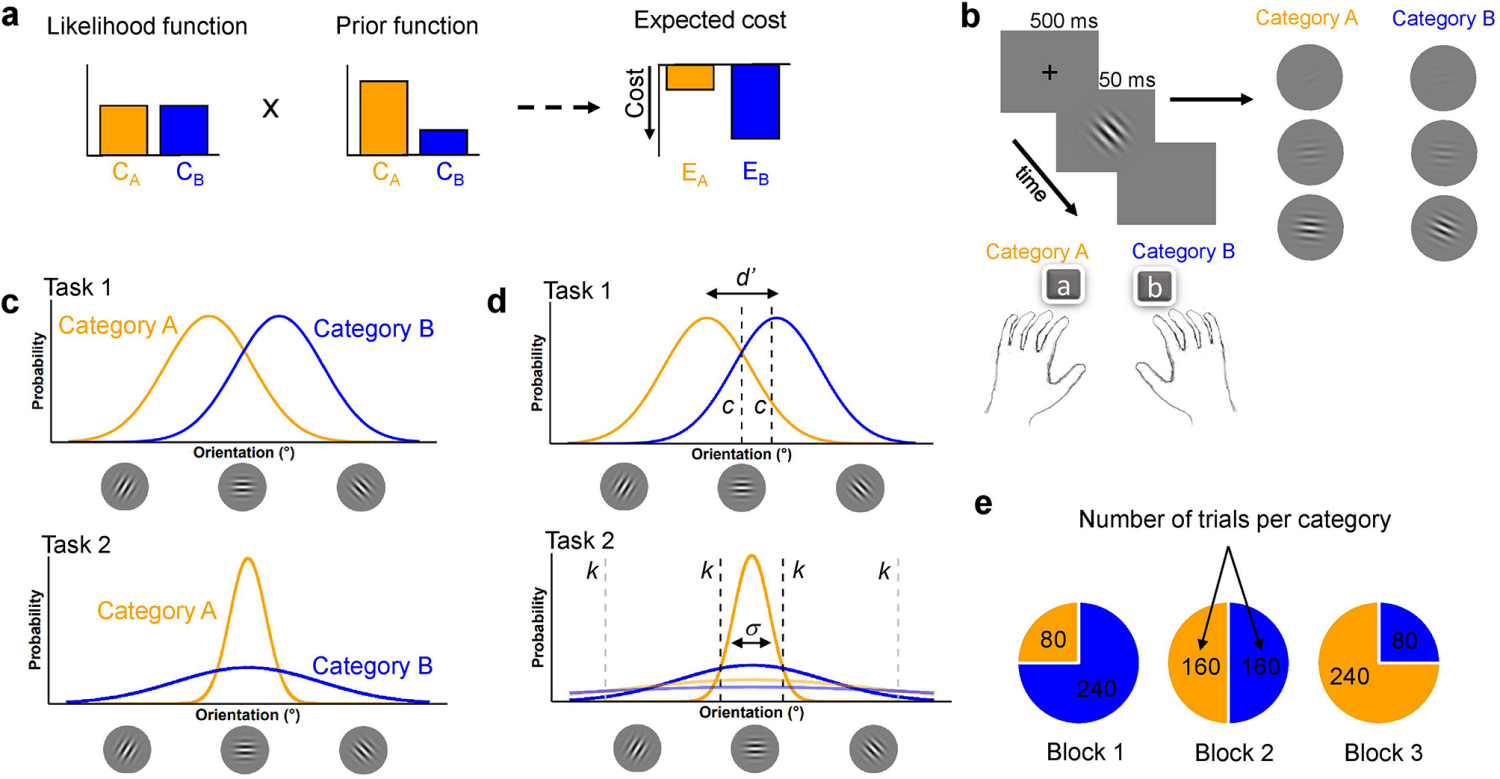
Theoretical framework and tasks. **(a)** Graphical depiction of how the Bayesian inference predicts the internal response and optimal decision criterion during a categorization task. An observer is deciding between two possible categories (Category A or Category B). We obtain the expected cost of each decision (E_A_ and E_B)_ by multiplying the sensory uncertainty and prior corresponding to each stimulus and then summing the costs associated with the two possible categories (here, we assume equal cost). **(b)** Illustration of the sequence of events within a trial in all experiments. For each trial, participants were asked to categorize the Gabor (Category A or Category B) depending on its orientation. The two sets of Gabor provide examples of possible orientations for each category, and different levels of contrast —from top to bottom, 0.004, 0.18, and 0.72. **(c)** Stimulus orientation distributions for each category in Task 1 (Experiment 1) and Task 2 (Experiment 2). **(d)** Illustration of the internal representation of the category distributions. In Experiment 1 (top graphic), *d’* represents the sensitivity or ability to separate the two categories, and c represents the adjustment of the decision criterion when the prior favors Category A. In Experiment 2, (bottom graphic), the distributions with vivid colors represent the internal representations of the categories when the sensory noise is low, and the faded colors when the sensory noise is high. *s* represents the internal noise, and *k* represents the decision boundaries, shifting outwards when the sensory noise is increasing. **(e)** Illustration of the number of trials for each category per block in Experiment 1. Each block contained 320 trials. Category B could appear with a probability of 75% (Block 1), 50% (Block 2), or 25% (Block 3). The block order was randomized across participants. In Experiment 2, each block contained the same number of trials (i.e., 320), and the two categories had an equal probability

In the effort to explain this variety of phenotypes, two related theoretical frameworks, Bayesian inference and predictive coding, suggested an underlying mechanistic account involving canonical processes of perceptual inference [6]. In both frameworks, perception is the outcome of inference processes that combine noisy external (sensory) information with internal models of the world. Bayesian inference is a computational framework in which sensory uncertainty (likelihood) and internal models (priors) are combined according to Bayes’ rule [7, 8] (Fig. 1a). Predictive coding provides a neural implementation of this integration process, which is not necessarily Bayesian [6, 9–11]. As an example of how these theoretical frameworks have explained perception, consider the following scenario: You see a large, shadowy figure during a night walk outside your home. If you live in some parts of North America, you know that bears live nearby. If you live in some parts of the Middle East, you know that boars live nearby. Because here the sensory information (likelihood) is uncertain, prior beliefs about the probability of encountering a boar or a bear would make a Bayesian observer more likely to categorize the shadowy figure as the animal that lives nearby than the one that lives on a different continent.

According to these theoretical frameworks, altered perception in autism arises from reduced use of prior beliefs. The Bayesian account postulates that difficulties in extracting prior information from the perceptual environment [12] or aberrant account of sensory evidence [13–15] could lead to an underuse of prior information in autism. The predictive coding view assumes an inflexibility to adjust prediction errors when sensory input deviates from expectations [16–18].

Despite the popularity of these views, which we collectively refer to as the altered integration hypothesis, evidence remains inconclusive [3, 19] and often depends on post hoc interpretations of results rather than the experimental manipulation of Bayesian components [3]. As a result, observed alterations in perceptual inference may reflect alterations in priors, sensory uncertainty, or the integration of the two.

The inconsistent findings could also stem from experimental shortcomings—such as inadequate prior learning attributable to compromised attentional or working memory capacities in autism [3, 19] (i.e., impaired learning of priors)—rather than a genuine reduced effect of priors [20, 21] (i.e., altered integration process). Moreover, studies often do not distinguish between different types of priors, such as natural (e.g., light from above [22]) vs. learned priors [18] or implicit (e.g., regression to the mean [23]) vs. explicit priors (e.g., base rate knowledge of wildlife in your area).

Overall, most Bayesian investigations have primarily focused on the effect of priors, thus failing to assess differences that may arise from the integration of sensory uncertainty. Thus, it remains unclear whether autistic individuals are truly impaired in prior integration or in the integration of other Bayesian components. Addressing these questions is critical to determine whether impairments in Bayesian inference constitute a core computational deficit in autism.

Here, we directly tested the altered integration hypothesis by systematically manipulating and testing the impact of each Bayesian component on perceptual decision-making. We used signal detection theory (SDT)—a standard model of decision-making and a special case of Bayesian inference—to estimate perceptual sensitivity and decision boundaries used to make categorical perceptual decisions. We manipulated priors (Experiment 1), and sensory uncertainty (Experiment 2) to directly assess the contribution of each Bayesian component to the decision boundary in autistic vs. non-autistic groups. This approach disentangled the effect of priors from sensory uncertainty using a unified experimental design. Importantly, explicit priors were given, allowing us to independently test and rule out differences between groups in prior knowledge. Stimulus contrast was manipulated to control for performance level, and the effectiveness of the prior manipulation was assessed to ensure task comprehension and motivation. Under these tightly controlled conditions, we found that autistic individuals (*N* = 49 in total) incorporate each Bayesian component into their perceptual decisions in a comparable manner to non-autistic controls (*N* = 77 in total), providing evidence against the altered integration hypothesis in autism.

## Experiment 1: the effect of prior knowledge on decision boundaries

In Experiment 1, we examined whether and how participants integrate prior knowledge during perceptual decision-making. Autistic and non-autistic individuals completed an orientation categorization task in which the stimulus on each trial was drawn from one of two categories, each characterized by a Gaussian distribution over orientation [24–26]. The orientation distributions were partially overlapping (see Task 1, Fig. 1c). To manipulate prior knowledge, we varied the base rates of the categories across different blocks (Fig. 1e). If participants were integrating priors, we hypothesized that the decision boundary would shift, favoring the category with the higher base rate (Task 1, Fig. 1d).

## Method

### Participants

Thirty-four adults diagnosed with autism (28 males and 6 females) and 49 non-autistic individuals (11 males and 38 females) participated in this experiment and received either monetary compensation (40 shekels/hour) or university credit compensation (3 credits/hour). Autistic participants were recruited from a reliable pool of participants routinely completing experiments for the Department of Special Education. The two groups recruited for the experiments matched in age (*t*(101.66) = 0.548, *p* = .585), the mean age was *m* = 26.58 years old, *se* = 0.90, for the autistic group, and *m* = 27.21, *se* = 0.71, for the non-autistic group. The IQ was evaluated using the Test of Non-Verbal Intelligence (TONI-4) measuring cognitive functioning without the interference of language deficits [27]. The two groups matched in IQ (*t*(60.61) = 0.82, *p* = .417), with a mean of *m* = 99.83, *se* = 11.47 for the autistic group, and *m* = 101.69, *se* = 9.91 for the non-autistic group. We used the Autistic Quotient (AQ) questionnaire to evaluate the participants’ autistic traits, and a t-test (*t*(64.47) = 6.30, *p* < .001) revealed that the autistic group had a significantly higher AQ, *m* = 26.89, *se* = 8.27, compared to the non-autistic group, *m* = 17.01 *se* = 6.80. We maintained a minimum 24 h-interval between consecutive experiments for each individual.

The autism diagnosis was confirmed through rigorous criteria, including the DSM-V, the Autism Diagnostic Interview (i.e., ADI-R52), and the Autism Diagnostic Observation Schedule (i.e., ASDOS-2). Moreover, all participants completed the Community Assessment of Psychic Experiences (i.e., CAPE) and AQ questionnaires, in their preferred language (Hebrew or English), either following the experimental phase or before the experiment, during the clinical assessment phase. We excluded non-autistic individuals with a history of epilepsy, neurological, psychiatric, or learning disorders, as well as those currently using psychiatric medications. We excluded individuals diagnosed with autism who have known genetic disorders (e.g., Down syndrome).

### Apparatus and stimuli

#### Apparatus

Stimuli were programmed in Matlab (The MathWorks, Inc., Natick, MA) with the Psychophysics Toolbox extensions, and were presented on a gamma-corrected 21-in CRT monitor (1280 × 960 resolution, 85-Hz refresh rate). Participants used the keyboard to respond.

#### Stimuli

Figure 1b illustrates the stimuli, experimental procedures, and tasks, based on Qamar et al. (2013), Adler and Ma (2018) and Denison et al. (2018). All stimuli were presented against a gray background (50 cd/m2). Each trial began with fixation (a black circle 0.2° of visual angle in diameter) for 500 ms, followed by the stimulus display for a duration of 50 ms. The stimulus was a sinusoidal grating with a two-dimensional Gaussian spatial envelope (i.e., Gabor patch), with *sd* = 0.325°, 85% contrast, and spatial frequency of 3 cycles per degree, presented at the center of the screen. In each trial, the orientation of the grating was randomly drawn from one of two Gaussian distributions, corresponding to the two stimulus categories (Fig. 1c). Following stimulus offset, participants reported both their category choice (Category A or B) and their level of confidence using a 4-point scale. This confidence rating scale ranged from high-confidence Category A to high-confidence Category B. The confidence data will be the focus of a separate paper. To manipulate the sensory uncertainty, we varied the stimulus contrast, randomly across trials, across seven fixed values (0.004, 0.016, 0.033, 0.093, 0.18, 0.36, 0.72).

#### Categories

Our experimental design incorporated continuous orientation distributions for each choice category, a critical feature enabling the separation of the participant’s sensory noise from their decision rule [26, 28]. Stimulus orientations were drawn from Gaussian distributions with means of *m*_A_ = 86°and *m*_B_ = 94° (tilts around vertical), both with standard deviations of *s*_A_ = *s*_B_ = 5° (see Fig. 1c, Task 1). The categories were partially overlapping, such that the maximum accuracy level was 80%. Participants were instructed to report which category they thought the stimulus belonged to on every trial, and they did not have any time limit to provide their answers.

### Procedure and design

#### Manipulation of prior knowledge

To manipulate priors, we varied the base rate of Category B (and conversely, Category A) across three blocks of trials. Two blocks had imbalanced base rates: one with a higher probability for Category A (B = 25% and A = 75%) and the other with a higher probability for Category B (B = 75% and A = 25%). The third block had balanced probabilities (B = 50% and A = 50%). See Supplementary Fig. 1a-c, for a depiction of the frequency of each orientation per category for each base rate block. The neutral block was always performed second. The order of the low and high blocks was counterbalanced between participants. Here, we expected a shift of decision boundary that favored the category with the high base rate (Fig. 1d, Task 1). Participants completed 960 experimental trials over approximately 50 min.

#### Manipulation verification

To ensure the comprehension of the main manipulations (i.e., base rate), a “check question” was randomly introduced during the experiment. Participants were asked to hypothetically gamble an amount of money on a category, ranging from 0 to 99 cents, on the chances of the next trial belonging to that category, and that the amount left would be automatically gambled on the other category. They were informed that their predictive performance would determine a monetary/credit bonus in addition to the original compensation.

#### Training

To ensure that all participants understood the task and manipulations, at the beginning of each experiment we conducted an extensive training phase on the categories and the confidence keys, then on the prior information at the beginning of each block (see Supplementary Methods).

### Data analyses

All analyses were performed on R version 4.2.2. Because confidence data was not the focus of the present study, we considered only the categorical response, collapsing across confidence keys.

To validate the manipulation of prior knowledge, we calculated the mean points gambled on category B in each base rate block for each participant. We then conducted a 2 × 3 mixed-design Analysis of Variance (ANOVA) with group (non-autistic, autistic) as the between-subject factor, category B base rate [75% (high for B), 50% (balanced), 25% (low for B)] as the within-subject factor, and points gambled on category B as the dependent variable.

For the main orientation categorization task, we first analyzed the raw data by calculating the probability of reporting category B across 16 binned orientation levels (-14, -12, -10, -8, -6, -4, -2, 0, 2, 4, 6, 8, 10, 12, 14) within each base rate condition. We then conducted a 3 × 16 × 2 mixed-design ANOVA with category B base rate and binned orientations as within-subject factors, and group as the between-subject factor on the probability of reporting B as the dependent variable.

To independently estimate perceptual sensitivity and decision boundary, we utilized the framework of standard signal detection theory (SDT). Sensitivity (d’) reflects the ability to discriminate between the two categories, while the decision criterion (c) indicates the decision bias participants employed to favor one category over the other. We then conducted a 7 × 3 × 2 mixed-design Analysis ANOVA with contrast (0.004, 0.016, 0.033, 0.093, 0.18, 0.36, 0.72) and category B base rate as within-subject factors, and group as the between-subject factor, on both d’ and c.

To assess overall adjustment of decision boundary to a change in category base rate, we computed the shift in *c* between biased base rate (75% and 25%) blocks *D*_criterion_ = c_75%_ - c_25%_. We conducted a 7 × 2 mixed-design ANOVA with contrast as within-subject factor and group as between-subject factor, on the *D*_criterion_.

To account for sensory uncertainty (the inverse of sensitivity) differences across and within subjects when assessing the effect of base rate on the decision boundary, we used an ideal observer analysis approach. We calculated the optimal criterion shift (c_opt_) based on the optimal bias (*β*), which was calculated for a range of d’ values (Eq. 1). *β* was derived from the base rate (*α*) condition (Eq. 2). The parameter α could take a value of *α* = 0.25 (low base rate) or *α* = 0.75 (high base rate).

1 $$\:c_{\text{opt}\:}=\frac{\text{l}\text{o}\text{g}\left(\beta\text{opt}\:\right)}{d{\prime\:}}$$

2 $$\:\beta_{\text{opt}}\:=\frac{(1-\alpha\:)}{\alpha\:\:}$$

Participants’ suboptimality *c*_error_ was estimated as the difference between a participant’s actual *c* and the corresponding *c*_opt_ based on their *d’* value, for each stimulus contrast. We conducted a 7 × 2 mixed-design ANOVA with contrast and group on the *c*_error_.

In all ANOVAs, significant effects were further investigated using paired and unpaired t-tests as Bonferroni corrections were applied to control for multiple comparisons. The effect sizes were calculated using partial eta square.

In addition, we used a t-test Bayes analysis to assess the evidence for differences between the autistic and non-autistic groups in sensitivity (*d’*), decision criterion (*D*_criterion_), and suboptimality (*c*_error_). Bayes factors (BF) were used to quantify the likelihood of the data occurring under assumptions of the alternative hypothesis (H1 = difference between the two groups) over the null hypothesis (H0 = no difference between the two groups). BF < 1 indicates that the data provide evidence in favor of H0. 1 < BF < 3 indicates weak evidence for H1. 3 < BF < 10 indicates moderate evidence for H1. BF > 10 indicates strong evidence for H1 [29].

We also performed linear-mixed effect models to confirm the main effects and interactions of category base rate and contrast on the sensitivity, decision criterion, and deviation from optimality.

Descriptions of analysis on linear mixed effect models, reaction time (RT) data and correlations between AQ and deviation from optimality can be found in Supplementary Methods, and the results are described in Supplementary Results and Supplementary Fig. 2a and 3a-b.

### Outlier removal

Participants with an accuracy below 0.6 at the three highest contrast levels and across blocks were excluded from all analyses. Participants demonstrating extreme deviation from an optimal observer (*c*_error_ > 50) were excluded from the optimality statistical analyses. Participants exhibiting an average reaction time that was three standard deviations away from their group’s mean were excluded from the reaction time analyses (Table 1).

**Table 1:** Description of the sample sizes in experiment 1, for the overall sample and in every statistical analysis, depending on the exclusion criteria based on participants’ performances: comprehension question, sensitivity, criteria, deviation from an optimal observer, reaction time, and correlation between the AQ and the criterion shift

	Overall *n*	Comprehension question	Sensitivity	Criteria	Optimality	rt	Correlation
Prior experiment	n_autistic_ = 34	n_autistic_ = 30	n_autistic_ = 31	n_autistic_ = 31	n_autistic_ = 30	n_autistic_ = 30	n_autistic_ = 23
	n_non−autistic_ = 49	n_non−autistic_ = 46	n_non−autistic_ = 46	n_non−autistic_ = 46	n_non−autistic_ = 45	n_non−autistic_ = 45	n_non−autistic_ = 40

## Results

### Prior manipulation verification

**Fig. 2:**
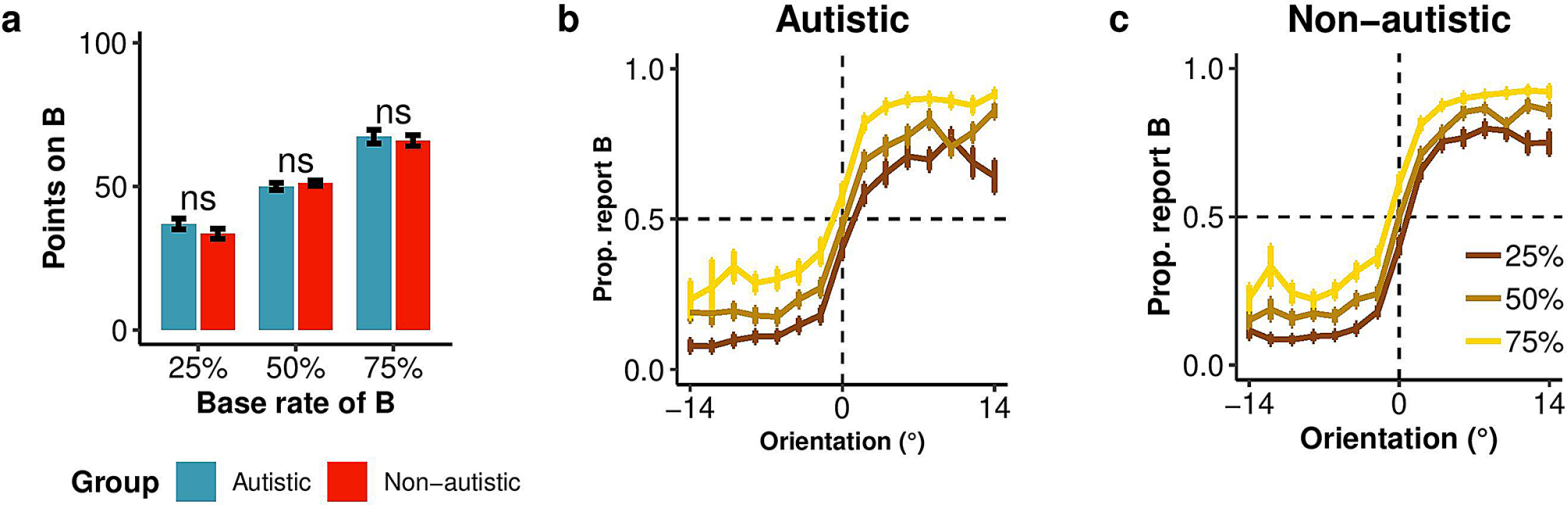
Task understanding and category report data for Experiment 1, prior manipulation. **(a)** Points gambled on category B as a function of base rate block. **(b**,** c)** Proportion of “Category B” responses as a function of orientation (x-axis) and Category B base rate block (line color) for the autistic and non-autistic groups. Data points show means across participants and error bars represent ± SE, per group of 30 autistic and 46 non-autistic participants in **(a)** and 31 autistic and 46 non-autistic participants in **(b**,** c)**. ns indicates no significant difference between groups evaluated using an unpaired t-test

Both autistic and non-autistic groups adjusted their gambling behavior in response to the base rate manipulation. An ANOVA on the average amount gambled on categories A and B showed a significant effect of base rate (Fig. 2a) (*F*(2, 144) = 122.38, *p* < .001, *h*_*p*_*²* = 0.63) with higher gambling points on the category with the higher base rate. We did not find a main effect of group (autistic vs. non-autistic, *F*(1, 72) = 0.92, *p* = .342, *h*_*p*_*²* = 0.01), nor an interaction between group and base rate (*F*(2, 144) = 0.52, *p* = .598, *h*_*p*_*²* < 0.01. These findings suggest that both groups of participants acquired prior knowledge to a similar extent.

### Categorization task

#### Category reports

Figure 2b-c illustrate, for each group, the probability of reporting Category B as a function of orientation. We observed a characteristic sigmoid shape with a higher probability of reporting Category B as the stimulus was oriented more clockwise (toward positive values). Category B reports increased, with an upward shift of the psychometric function, when there was a high base rate for Category B, and decreased (downward shift) when there was a low base rate for Category B. This shift of probability was supported by an ANOVA, showing a main effect of block on the probability of reporting Category B. *F*(1.45, 109.08) = 62.15, *p* < .001, *h*_*p*_*²* = 0.45. Overall, the pattern of results is comparable across groups, with no significant main effect of group, *F*(1, 75) = 0.85, *p* = .359, *h*_*p*_*²* = 0.01, or interaction between group and block, *F*(1.45, 109.08) = 0.98, *p* = .355, *h*_*p*_*²* = 0.01.

#### Perceptual sensitivity

**Fig. 3:**
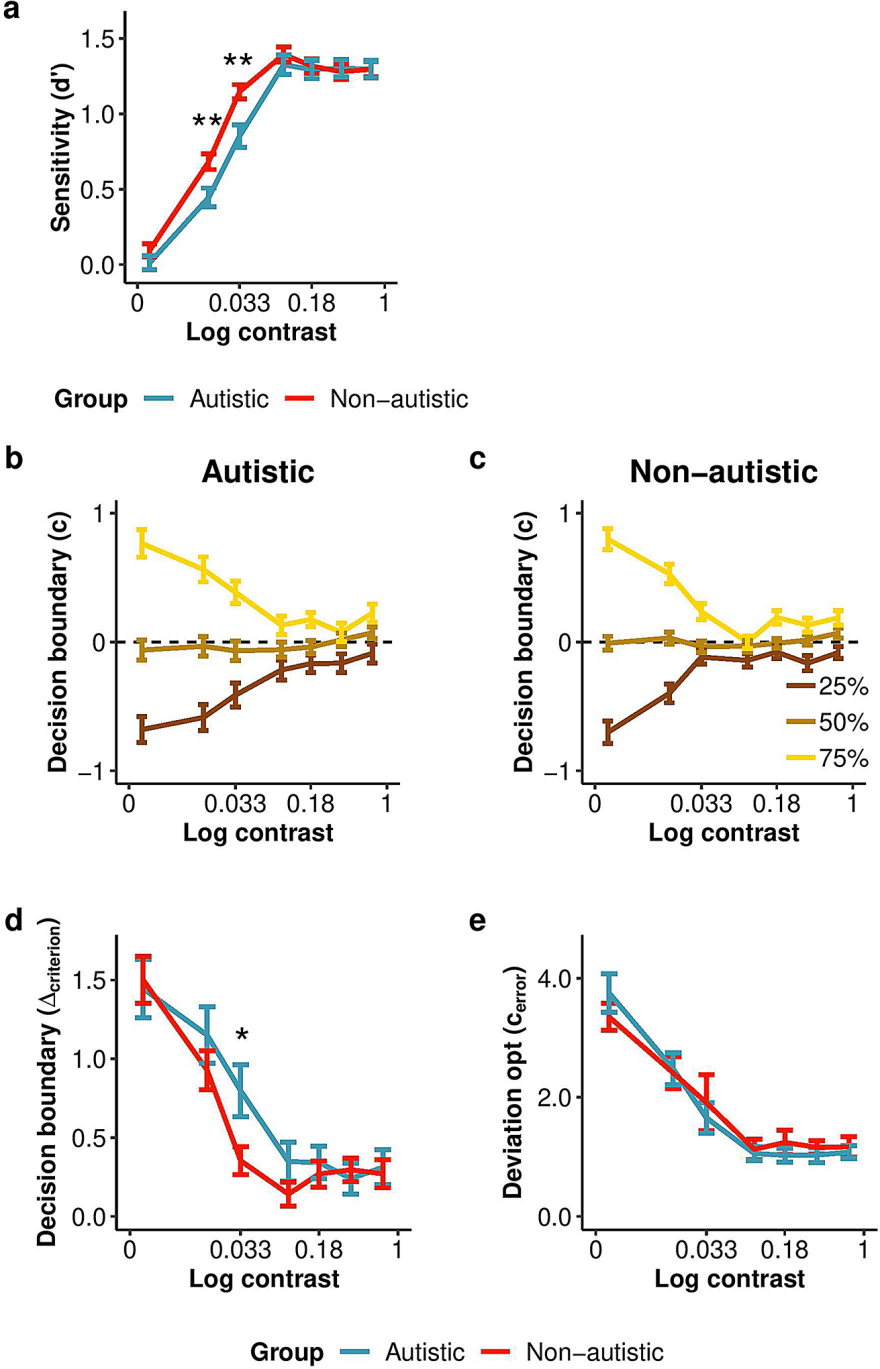
Sensitivity, decision boundary, and optimal observer analyses for Experiment 1, prior manipulation. **(a)** Sensitivity (*d’*) for each group as a function of contrast and across base rates. Note that in all experiments, the relatively low sensitivity in both groups, even when contrast is high, is due to the limit of a maximum of 80% correct in these tasks. **(b**,** c)** Decision criterion (*c*) as a function of contrast for the three base rate blocks for the autistic and non-autistic groups. The base rate legend gives the probability for category B to appear. **(d)** Difference between criterion shifts in biased (25% and 75%) base rate blocks (*D*_criterion_) for each group as a function of contrast on a log scale. **(e)** Deviation of criterion shift from optimality (*c*_error_) as a function of contrast. Participants showed an increase in deviation from an optimal criteria adjustment as contrast decreased, with no difference between autistic and non-autistic groups in the degree to which the criterion was suboptimal. Data points show means across participants and error bars represent ± SE. The asterisks represent the group difference evaluated using unpaired t-tests, *p ≤ .05, **p ≤ .01. The sample size constituted 31 autistic and 46 non-autistic participants in **(a)**, **(b)**,** (c)** and **(d)**, and 30 autistic and 45 non-autistic participants in **(e)**

Perceptual sensitivity for orientation categorization increased with contrast for both groups, confirming that the manipulation of sensory uncertainty was effective. An ANOVA on sensitivity (*d’*) showed a significant main effect of contrast level, *F*(6, 450) = 151.82, *p <* .001, *h*_*p*_*²* = 0.67 (Fig. 3a). There was no main effect of group, *F*(1, 75) = 2.20, *p* = .142, *h*_*p*_*²* = 0.03. However, there was a significant interaction between group and contrast level, *F*(6, 450) = 2.29, *p* = .034, *h*_*p*_*²* = 0.03. Post-hoc t-tests revealed that this interaction stemmed from greater sensitivity in the non-autistic group compared to the autistic group at two contrast levels: 0.016 (*t*(198) = 2.92, *p* = .004) and 0.033 (*t*(162) = 3.34, *p* = .001). The effects of base rate blocks and the interaction between base rate and contrast levels are detailed in the Supplementary Results. The t-test Bayes factor estimating the likelihood of the alternative hypothesis assuming a difference in sensitivity between groups (H1) over the null hypothesis assuming no difference between groups (H0) provided weak evidence for the alternative hypothesis (BF_10_ = 1.59 ± 0.01%). Whereas some have proposed that greater sensory precision in autism reduces the use of prior information, here we found, if anything, reduced perceptual sensitivity for the autistic group.

#### Decision boundaries

Decision boundaries determine whether a stimulus orientation will be categorized as coming from category A or B. To quantify how the decision boundary shifts with prior information about category base rate, we computed the decision criterion for each base rate block and contrast level (Fig. 3b-c) and calculated the criterion shift (*D*_criterion_) as the shift between the two biased base-rate conditions (25% and 75%). An ANOVA showed a significant effect of contrast level on *D*_criterion_, *F*(6, 450) = 60.49, *p* < .001, *h*_*p*_*²* =0.45, indicating that the criterion shift increased as contrast decreased (Fig. 3d), consistent with the Bayesian prediction of greater reliance on the prior when sensory information is more uncertain. There was no effect of group, *F*(1, 75) = 0.99, *p* = .321, *h*_*p*_*²* < 0.01. The interaction between group and contrast level was significant, *F*(6, 450) = 2.12, *p* = .05, *h*_*p*_*²* = 0.03, and post-hoc t-tests revealed that at contrast 0.033, autistic participants showed a significantly greater criterion shift than non-autistics, *t*(46.8) = 2.37, *p* = .022. The Bayes factor (BF_10_ = 0.38 ± 0.05%) supported the evidence for the null hypothesis assuming no difference in criteria shift between groups. Overall, autistic and non-autistic participants adjusted their decision criterion in response to the prior manipulation.

#### Suboptimality

To assess criterion shift while controlling for variations in sensitivity, we compared the observed *c* shift to the shifts expected for an optimal observer. For each individual, at each contrast level and biased base rate condition, we calculated the deviation from optimality (*c*_error_) values by subtracting the observed criterion from the optimal criterion (see Methods, Data analyses). The further from zero, the more participants’ criterion deviated from an optimal observer. An ANOVA on the *c*_error_ showed a significant main effect of contrast level on *c*_error_, *F*(6, 438) = 29.61, *p* < .001, *h*_*p*_*²* = 0.29 (Fig. 3e). Participants demonstrated larger *c*_error_ as contrasts decreased, indicating a more suboptimal shift when sensory evidence was weaker. Critically, there was no effect of group, *F*(1, 73) = 0.04, *p* = .851, *h*_*p*_*²* < 0.01, and no interaction between group and contrast level, *F*(6, 438) = 0.41, *p* = .873, *h*_*p*_*²* < 0.01. The results of the ANOVA were supported by the Bayes factor (BF_10_ = 0.07, ± 0.27%), providing strong evidence for H0 (i.e., no difference in suboptimality between groups).

The linear-mixed effect models performed on the sensitivity, the decision boundaries, and the deviation from optimality were consistent with the AVNOA findings. The results are detailed in Supplementary Results, Supplementary Tables 1, and Supplementary Table 2.

These results indicate that, when perceptual sensitivity is taken into account, autistic individuals adjust their criteria in the same suboptimal manner as non-autistic individuals: both groups deviate more from an optimal observer as sensory evidence decreases. This finding contradicts the altered integration hypothesis in autism.

### Experiment 2: the effect of sensory uncertainty on decision boundaries

The results of Experiment 1 demonstrate that autistic participants adjusted their decision criterion in response to changes in prior information in a typical though suboptimal manner. Suboptimality in this task could arise from inadequate use of priors, or inadequate assessments of the observer’s own sensory uncertainty, and the contributions of these two factors could differ across autistic and non-autistic participants. To distinguish between these possibilities, in Experiment 2 we asked whether autistic individuals could adjust their decision boundary to take into account variations in their own sensory uncertainty, separate from prior manipulation. This experiment thus isolates the likelihood function, to determine whether and how autistic participants account for changes in their own sensory uncertainty during perceptual decision-making.

Because in Experiment 1 (Task 1), participants had no incentive to adjust their categorical decision boundaries in response to changes in sensory uncertainty alone [30], in Experiment 2, to specifically tap the contribution of sensory uncertainty to decision-making, we used a task in which changes in sensory uncertainty alone required an adjustment in decision rules to maximize task performance. Participants performed an embedded category task [24–26] (see Fig. 1c, Task 2) in which they were asked to distinguish between a broad category of orientation and a narrow one. Sensory uncertainty was manipulated by varying the stimulus contrast trial by trial. Integration of sensory uncertainty information in the decision-making process would be evident if decision boundaries shifted outward as sensory uncertainty increased (Fig. 1d, Task 2).

## Method

### Participants

Participants’ recruitment process was the same as in Experiment 1. Thirty-four adults diagnosed with autism (27 males and 7 females) and 44 non-autistic individuals (11 males and 33 females) participated in this experiment. Among these participants, 19 autistic and 16 non-autistic participants previously participated in Experiment 1.

### Apparatus, stimulus, procedure and design

Apparatus, Stimulus, Procedure and Design were the same as in Experiment 1, except for the following changes. Instead of Task 1, an embedded categorization task (Task 2) was used in Experiment 2. In this task, stimulus orientations were drawn from Gaussian distributions with identical means, *m*_A_ = *m*_B_ = 0 ° (horizontal), but differing standard deviations, *s*_A_ = 3 ° and *s*_B_ = 12 ° (see Fig. 1c, Task 2, and Supplementary Fig. 1d, for a depiction of the frequency of each orientation per category).

In Experiment 2, there was no explicit manipulation between blocks and, therefore, no need to verify understanding of the experimental manipulation, yet, to maintain consistency and motivation we used the “check question” from Experiment 1.

Preliminary data indicated that Task 2 was more susceptible to noise. Therefore, participants performed two separate sessions of 960 trials each, with a minimum 24-hour gap between them.

### Data analysis

To investigate how the manipulation influence participants’ behavior, we conducted a mixed-design ANOVA with 3 factors: (1) group (non-autistic, autistic), (2) contrast (0.004, 0.016, 0.033, 0.093, 0.18, 0.36, 0.72), and (3) orientation (-14, -12, -10, -8, -6, -4, -2, 0, 2, 4, 6, 8, 10, 12, 14), on the probability to report category B.

We used a modified SDT model to estimate sensitivity and decision boundaries for the embedded category task. In this task, the two category distributions have the same mean orientation of 0°, but different standard deviations, *s*_A_ = 3° and *s*_B_ = 12°. The observer’s estimated orientation is subject to additional internal noise, which depends on their perceptual sensitivity, *σ*, at each contrast. The standard deviation for the internal measurement distribution of each category across trials, combining external and internal noise, is then as displayed in Eq. (3).

3 $$\:{\sigma}_{\text{c}\text{a}\text{t}}=\sqrt{{{s}_{\text{c}\text{a}\text{t}}}^{2}+{{\sigma}_{\text{s}\text{e}\text{n}\text{s}}}^{2}}$$

In the embedded category task, the observer sets decision boundaries *k* to distinguish between the narrow category A and the broad category B. For the purpose of model fitting, we assume these boundaries to be symmetrical around zero degrees and stable across trials (Fig. 1d, Task 2). Then the probability of reporting category A for a given stimulus category *C*_cat_ with orientation noise *σ*_cat_ is given by the area of the internal measurement distribution across trials that falls between the decision boundaries.

The probability of reporting Category B for a given stimulus category is 1 minus that number.

We estimated *σ*_sens_ and *k* from the data at each contrast level using the proportions of the participant’s category reports across trials, according to Eq. (4).

4 $$\:p\left({r}_{\text{A}}|{C}_{\text{c}\text{a}\text{t}},{\sigma\:}_{\text{c}\text{a}\text{t}}\right)={\int\:}_{-k}^{k}\mathcal{N}\left(0,{\sigma\:}_{\text{c}\text{a}\text{t}}\right)$$

To do so, we took advantage of the fact that participants have the same internal noise and set of decision boundaries across both categories, and the means and standard deviations of the stimulus distributions are known. We first used an optimization procedure (*fmincon* in MATLAB), with a lower boundary of 0 and no upper boundary, to estimate what value of *σ*_sens_ was most consistent with a single *k* across both categories, given the reports. We then calculated *k* using the fitted value of *σ*_sens_. We confirmed that this procedure correctly recovered *σ*_sens_ and *k* values from simulated data.

We conducted mixed-design ANOVAs on *σ*_sens_ and *k* with the following factors: (1) contrast (0.004, 0.016, 0.033, 0.093, 0.18, 0.36, 0.72) and (2) group (non-autistic, autistic).

To control for any variation in perceptual sensitivity across participants, we calculated the optimal decision boundary *k*_opt_ using the participant’s estimated *σ*_sens_ combined with the stimulus standard deviations to give *σ*_A_ and *σ*_B_ (Eq. 5),

5 $$\:{k}_{\text{o}\text{p}\text{t}}=\pm\:\frac{{\sigma}_{\text{A}}^{2}{\sigma}_{\text{B}}^{2}}{{\sigma}_{\text{B}}^{2}-{\sigma}_{\text{A}}^{2}}\sqrt{2\text{log}\frac{{\sigma}_{\text{B}}}{{\sigma}_{\text{A}}}}$$

The optimal boundary *k*_opt_ lies at the crossing points of the internal measurement distributions for the two categories and maximizes performance across trials. We used the positive *k* values for all analyses.

We then estimated each participant’s degree of suboptimality (*k*_error_) by comparing *k* to the corresponding *k*_opt_ for each contrast level. We performed a mixed-design ANOVA with two factors: (1) contrast (0.004, 0.016, 0.033, 0.093, 0.18, 0.36, 0.72) and (2) group (non-autistic, autistic) on the *k*_error_.

Significant effects from the ANOVAs, Bayes factor, effect sizes, correlations between the AQ and the deviation from an optimal observer, linear-mixed effect models, and RT analyses were conducted the same way as for Experiments 1. (see Supplementary Methods, Results, Fig. 2b, and Fig. 3c for the linear-mixed effect model, correlation and RT results).

#### Outlier removal

Participants with an accuracy below 0.6 at the three highest contrast levels and across blocks were excluded from all analyses. Additionally, participants showing extreme criterion shift (*k* > 100) or a estimated uncertainty (*s* > 100) were removed from all analyses. Participants demonstrating extreme deviation from an optimal observer (*k*_error_ > 35) were excluded from the optimality statistical analyses. The outlier detection for the RT was the same as in Experiment 1. The sample size of each analysis is displayed in Table 2.

**Table 2:** Description of the sample sizes in experiment 2, for the overall sample, and in every statistical analysis, depending on the exclusion criteria based on participants’ performances: sensitivity, criteria, deviation from an optimal observer, reaction time, and correlation between the AQ and the criterion shift

	Overall *n*	Sensitivity	Criteria	Optimality	rt	Correlation
Likelihood experiment	n_autistic_ = 34	n_autistic_ = 27	n_autistic_ = 27	n_autistic_ = 27	n_autistic_ = 24	n_autistic_ = 23
	n_non−autistic_ = 44	n_non−autistic_ = 40	n_non−autistic_ = 40	n_non−autistic_ = 38	n_non−autistic_ = 39	n_non−autistic_ = 37

## Results

### Category reports

**Fig. 4:**
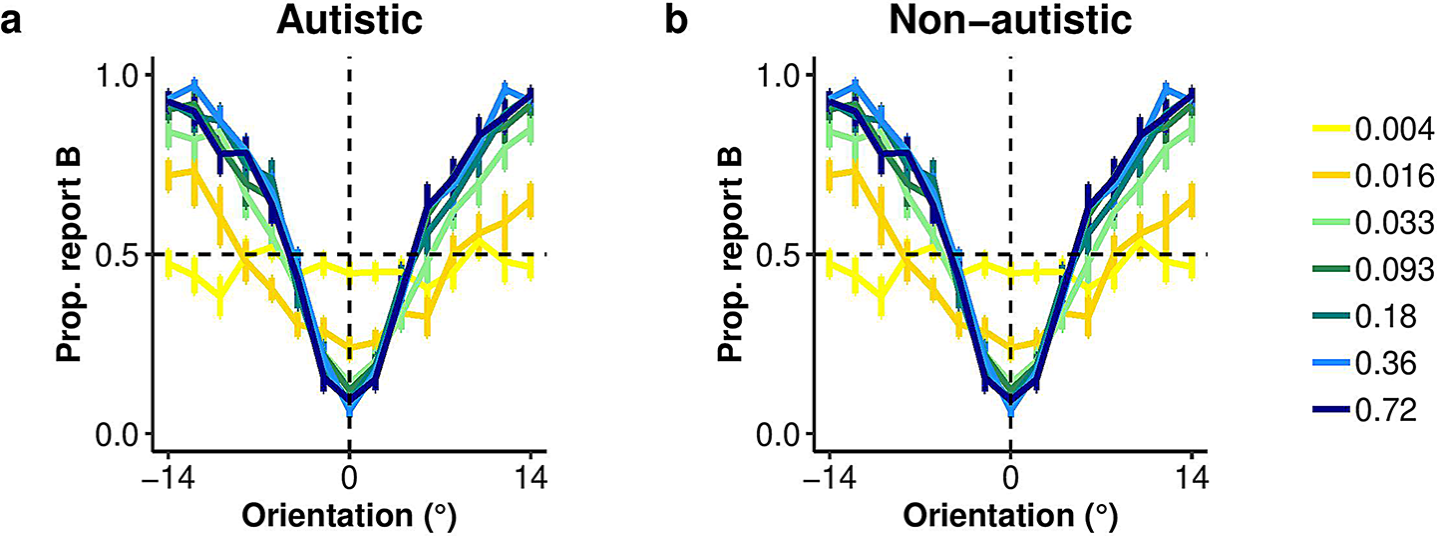
Category report data for Experiment 2, sensory uncertainty manipulation. **(A**,** B)** Illustration of the proportion of reporting Category B as a function of orientation (x-axis) and contrast levels (line color) for the autistic (*n* = 27) and non-autistic (*n* = 40) groups. Data points show means across participants and error bars represent ± SE

Figure 4a-b illustrates, for each group, the probability of reporting Category B as a function of orientation. Using the embedded category task (Experiment 2), the probability of reporting Category B (with the wider distribution) increased as the stimulus was oriented away (clockwise or counterclockwise) from horizontal (0°; see Fig. 1d, Task 2). For both groups, category reports became more sensitive to stimulus orientation as contrast increased. These observations were supported by the ANOVA showing a main effect of contrast on the probability to report Category B, *F*(3, 189.11) = 31.47, *p* < .001, *h*_*p*_*²* = 0.33. The main effect of group (*F*(1, 63) = 0.07, *p* = .793, *h*_*p*_*²* < 0.01) and the interaction between group and contrast (*F*(3, 189,11) = 2.26, *p* = .083, *h*_*p*_*²* = 0.04) were not significant.

#### Perceptual sensitivity

**Fig. 5:**
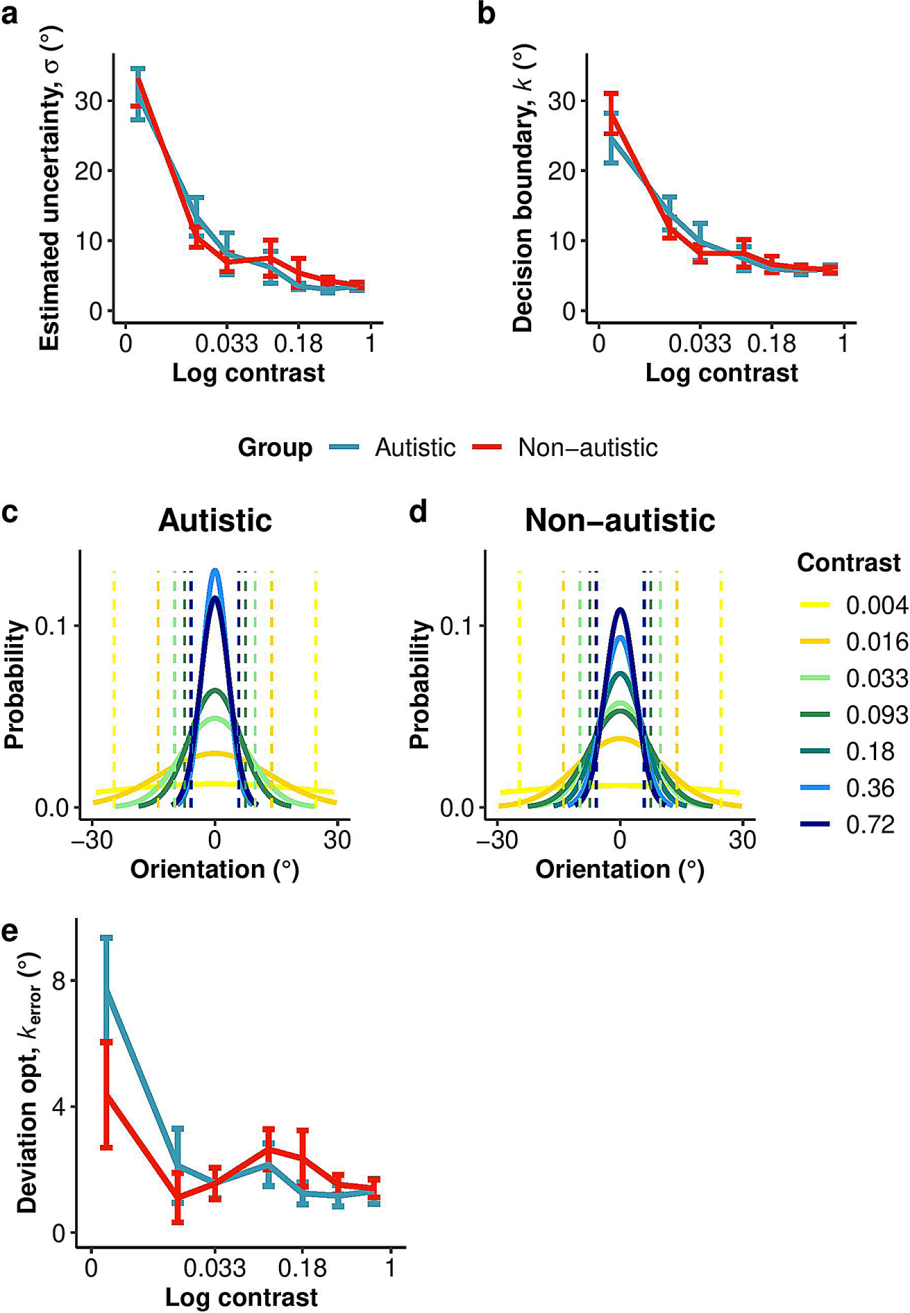
Sensitivity, decision boundary, and optimal observer analyses for Experiment 2, sensory uncertainty manipulation. **(a)** Sensory uncertainty was evaluated by fitting the data with an SDT-style model adapted to the embedded category task. The fitted standard deviation, *s*, provided an estimate of sensory uncertainty. A higher value indicates more sensory uncertainty compared to a lower value. **(b)** Category boundaries *k* were estimated from the same model and assumed to be symmetrical about zero degrees; the positive value is shown. **(c**,** d)** Probability of the category distributions for each level of contrast. The solid lines represent the precision of the distribution, with the sensory uncertainty (*s*) as standard deviation of the category representations. The dashed lines represent the averaged decision boundaries (*k*) for each level of contrast **(e)** Deviation from optimality *c*_error_ as a function of contrast. Participants showed a larger deviation from the optimal decision boundaries as contrast decreased. Data points show means across participants and error bars represent ± SE. The sample size was 27 autistic and 40 non-autistic participants in **(a-d)**, and 27 autistic and 38 non-autistic participants in **(e)**

Increasing contrast led to lower sensory uncertainty, as estimated by an SDT-style model adapted to the embedded category task (see Methods), consistent with the expected effect of contrast in improving orientation information. The ANOVA conducted on the parameter *σ* of the model, which provided an estimate of sensory uncertainty, revealed a significant main effect of contrast level *F*(6, 390) = 46.03, *p* < .001, *h*_*p*_*²* = 0.42), confirming that the manipulation of contrast-induced a change in sensory uncertainty (Fig. 5a). There was no significant difference between group *F*(1, 65) = 0.07, *p* = .794, *h*_*p*_*²* < 0.01), nor an interaction between group and contrast *F*(6, 390) = 0.39, *p* = .887, *h*_*p*_*²* < 0.01. These results were supported by the Bayes factor (BF_10_ = 0.11 ± 0.15%) providing evidence for the null hypothesis (i.e., no difference in sensitivity between groups). These findings suggest that both groups exhibited similar changes in sensitivity in response to the contrast manipulation in the embedded category task.

#### Decision boundaries

Participants’ categorical decision boundaries depended on contrast (Fig. 4a-b). Both groups shifted their categorical decision boundaries outward as sensory uncertainty increased, the qualitative pattern expected from a Bayesian observer (Fig. 5b-d). The ANOVA conducted on the *k* parameter of the model, which provides an estimate of the category boundaries, revealed a significant main effect of contrast *F*(6, 390) = 38.56, *p* < .001, *h*_*p*_*²* = 0.37, indicating that the participant’s decision boundaries were sensitive to the sensory uncertainty manipulation. Notably, there was no significant effect of group (*F*(1, 65) = 0.03, *p* = .858, *h*_*p*_*²* < 0.01), nor an interaction between group and contrast, *F*(6, 390) = 0.59, *p* = .741, *h*_*p*_*²* < 0.01). The Bayes factor BF_10_ = 0.11 ± 0.16%) provided strong evidence for the null hypothesis (i.e., no difference in criteria shift between groups), supporting the ANOVA’s results. These results indicate that both groups adjusted their decision boundaries similarly in response to changes in sensory uncertainty.

#### Suboptimality

Next, we asked how much the decision boundary shifts in autistic and non-autistic participants deviated from those of an optimal Bayesian observer (Fig. 5c). The ANOVA conducted on *k*_error_ revealed a significant main effect of contrast *F*(6, 378) = 11.06, *p* < .001, *h*_*p*_*²* = 0.15, with a greater deviation from the optimal decision boundaries when contrast was lower. There was no significant difference between groups, *F*(1, 63) = 0.16, *p* = .688, *h*_*p*_*²* < 0.01) and no significant interaction between group and contrast, *F*(6, 378) = 2.10, *p* = .053, *h*_*p*_*²* = 0.03. These results were supported by the Bayes factor (BF_10_ = 0.13 ± 0.13%) providing strong evidence for the null hypothesis (i.e., no difference in suboptimality between groups). These results show that during perceptual decision-making, autistic individuals take sensory uncertainty into account similarly to the non-autistic group.

The linear-mixed effect model results on the sensitivity, decision boundaries, and deviation from optimality supported all these findings (see Supplementary Results).

## Discussion

We conducted a series of experiments to investigate Bayesian inferences in visual perceptual decision-making in autistic individuals and non-autistic controls. In these experiments, participants performed an orientation categorization task, while we separately manipulated category base rate and sensory uncertainty. In a Bayesian framework, these manipulations would induce changes in each Bayesian decision component: prior knowledge, and sensory uncertainty respectively. This study reveals that, despite some differences in sensitivity to orientation information, the autistic group adjusted their decision criterion to accommodate variations in priors and sensory uncertainty, in a manner comparable to the suboptimal adjustments of the non-autistic group. Autistic participants are intact in incorporating each Bayesian component into their perceptual decisions. These results prompt a reevaluation of the altered integration hypothesis.

### Perceptual priors

The altered integration hypothesis, despite a lack of direct evidence, remains prevalent in autism research [12, 20, 21, 30, 31]. The most straightforward method to quantitatively assess prior integration is to test the effect of base rate probability on decision criterion. To date, only one study, Skewes and Gebauer (2016), using a categorical localization task of auditory stimuli, has directly addressed this in autism. They showed that autistic individuals adjusted their criterion to a lesser extent compared to non-autistics to favor the location category with the higher base rate probability. Notably, their study lacked explicit instruction regarding base rate manipulation or an independent test of it, leaving it unclear whether group differences were due to altered integration or simply reduced prior learning. In our study, by using explicit base rate instruction and an independent measure of prior knowledge—the gambling questions—we ensured that prior knowledge was consistent across groups. Additionally, by varying stimulus contrast levels, we tested for prior integration across various levels of sensory uncertainty within the same individual. Our results reveal that, after controlling for possible group differences in perceptual sensitivity and task knowledge, autistic individuals integrate priors to the same extent as non-autistic individuals.

In the prior experiment, we observed reduced sensitivity in the autistic group at certain contrast levels. Although enhanced sensory processing, particularly enhanced estimation of sensory reliability, has been proposed as an explanation for atypical perception in autism, evidence for enhanced contrast sensitivity remains limited. While some studies report no differences in contrast sensitivity in autism [2, 32, 33], others report reduced sensitivity [34, 35]. Our study aligns with those findings by showing some reduction in contrast sensitivity in Experiment 1, but similar sensitivity in Experiment 2.

### Sensory uncertainty

The altered integration view suggests that enhanced sensory evidence, or lower sensory uncertainty, could be an alternative to the reduced priors account. For a Bayesian observer, reduced priors and lower sensory uncertainty are mathematically indistinguishable from decision outcomes alone [13]. Lower sensory uncertainty does not necessarily entail higher performance but rather a subjective representation of reduced sensory uncertainty. To address the hypothesis that autistic individuals use information about their own sensory uncertainty in an atypical fashion, we employed an embedded category task to assess whether participants adjust their decision criterion based on sensory uncertainty per se. If autistic individuals have an atypical representation of sensory uncertainty, this would be reflected in their decision criterion. However, our results show a similar pattern of criterion adjustment in both groups, revealing that autistic individuals have a sensory uncertainty representation similar to non-autistic individuals.

### Limitations

Here we used perceptual categorization of orientations, which enabled us to directly quantify and compare the prediction of the altered Bayesian integration hypothesis. However, altered Bayesian inference in autism may occur at different levels of processing, including very early sensory processing, such as basic detection, as well as higher-level decision-making and social behavior (e.g., faces).

We used explicit manipulation of prior knowledge, which enabled us to focus on the integration process per se, however, the difference in the use of prior might be specific to learned priors and related to the rate and flexibility of learning and updating [23, 36]. Therefore, future research could focus on differences in perceptual prior learning and updating, or on implicit perceptual inferences that do not involve an explicit perceptual decision. Additionally, examining the role of attentional and working memory capacities may provide insights into how autistic individuals process sensory information.

Our clinical population was high-functioning adults, thus, it is possible that behavioral strategies and other processes during development may help to compensate for differences that may emerge in Bayesian perception during early childhood. Future studies should address these issues by testing Bayesian inference using various stimulus type (e.g., social stimuli), prior acquisition methods (e.g., implicit vs. explicit), and populations. Finally, a potential limitation of our study is the difference in sex ratio between the autistic and the non-autistic groups. However, as there are no reports of sex differences in orientation discrimination tasks, the difference in sex ratio cannot account for our findings.

## Conclusions

Through two experiments, this study provides a systematic investigation of all Bayesian components of perceptual decision inference. The findings reveal that autistic individuals take into account prior knowledge and sensory uncertainty in a manner similar to non-autistic individuals, though both groups exhibit suboptimal behavior. These results challenge the current views of altered integration in perception and sensory processing in autism.

The demonstration that autistic individuals are capable of typical integration of Bayesian components has important implications for developing more targeted interventions and support strategies aimed at enhancing perceptual and cognitive functioning in autistic individuals. Specifically, the findings show that given accurate and explicit knowledge, high-functioning autistic individuals can use contextual information in a typical manner. These findings have direct occupational implications.

## Electronic supplementary material

Below is the link to the electronic supplementary material.

Supplementary Material 1

## Data Availability

The datasets supporting the conclusions of this article will be available in the Open Science Framework (OSF) repository: https://osf.io/mq4kn/.
