# Supplementary material for: Suboptimal but intact integration of Bayesian components during perceptual decision-making in autism
Source: Mol Autism. 2025 Jan 13;16:2. doi: 10.1186/s13229-025-00639-3 (PMC11731163; doi:10.1186/s13229-025-00639-3)
Supplement: Supplementary file 1 — Supplementary Material 1 [file 13229_2025_639_MOESM1_ESM.docx]

**Supplementary Information**

**Supplementary Methods**

**
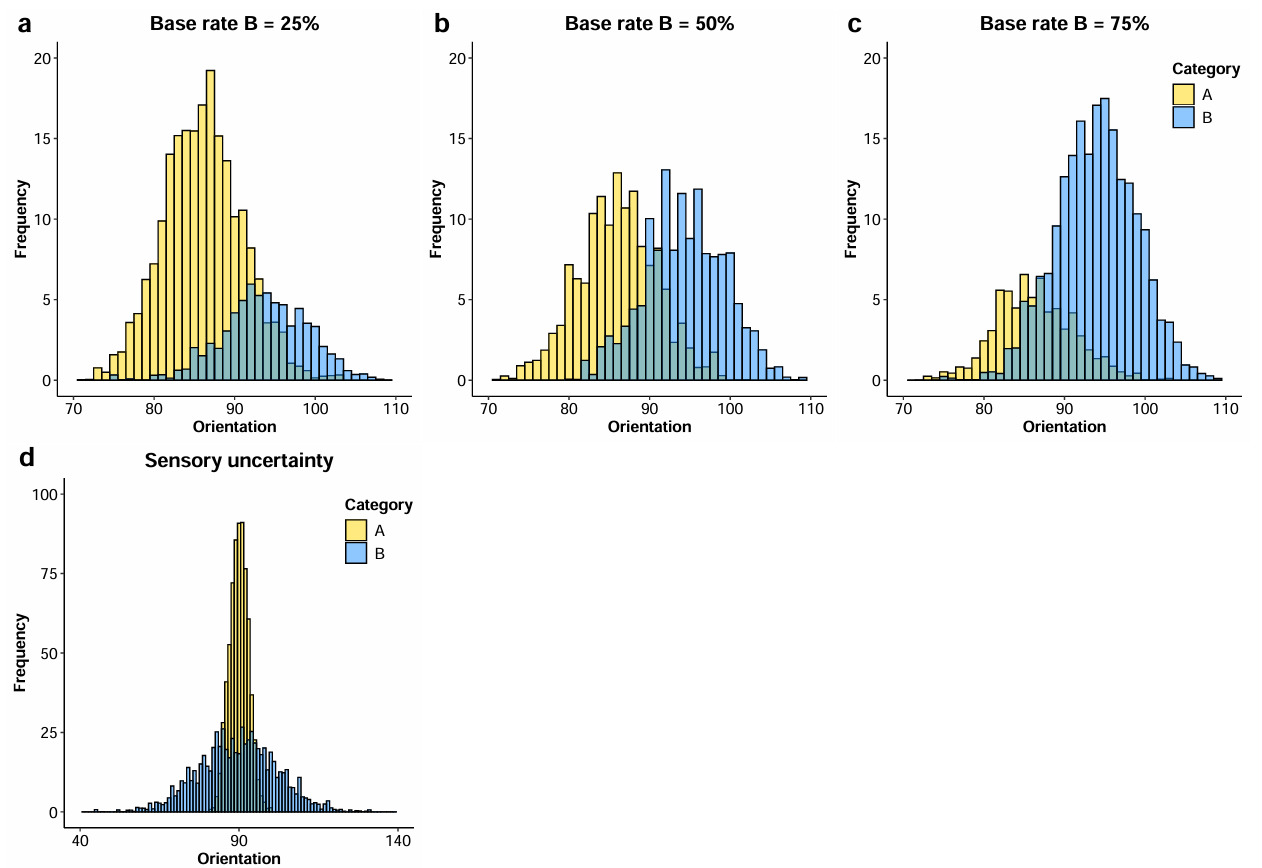
**

**Supplementary Figure 1. Illustration of the frequency of the orientations for each category.** In Experiment 1, the frequencies varied between blocks, depending on the base rate condition. The base rate for Category B could be 25% **(a)**, 50% **(b)**, or 75% **(c)**. In Experiment 2, the base rate stayed the same between blocks **(d)**.

**Task training**

*Category training*. At the start of each experiment, participants were shown a printed graphic similar to **Figs. 1b-c**, which explained the generation of stimuli from specific distributions. Subsequently, to ensure participants were acquainted with the stimulus distributions, they underwent category training, first with each category separated, then with Category A and Category B combined with equal probabilities. In total, the practice consisted of 40 trials: 10 trials per category and 20 trials for the combined practice). On each trial, the stimulus orientation was drawn from the corresponding stimulus distribution (**Fig. 1c**). The stimulus presentation duration was 300 ms and contrast was 100%. Following the participant’s response, a text message displaying their chosen category was presented, along with auditory correctness feedback (i.e., high pitch sound indicating a correct answer, and a low pitch sound indicating an incorrect answer).

*Confidence training*. Participants also completed a brief confidence training block to familiarize them with the key mappings used to report both their category choice and their confidence in each trial. We provided them with a printed graphic illustrating the key layout, indicating that participants needed to press one of eight buttons to indicate both category choice (A or B) and confidence level (on a 4-point scale). The confidence levels were labeled as “High,” “Medium-High,” “Medium-Low,” and “Low.” Trial-to-trial feedback consisted of a message confirming which category and confidence level they had reported, without any correctness feedback.

*Base rate training*. In Experiment 1, we introduced the participants to the conditions of each experimental block (e.g., base rate) with a verbal explanation followed by a practice session of 40 trials during which they reported both the category and their confidence level. Immediately after their response, the screen displayed the chosen category, along with a feedback sound. After reaching an accuracy of around 70%, reflecting that they were familiar enough with the categories, the response keys, and the block conditions, participants proceeded to complete the block of 280 test trials.

Throughout the experiment, participants did not receive trial-to-trial feedback to ensure that their decision boundaries were internally generated and not learned from correctness feedback. However, after every 50 trials, they were shown the percentage of trials they had correctly categorized to maintain motivation. In Experiment 2, participants were also provided with information on the points earned during the last 50 trials and the points accumulated over the experiment.

**Data analysis**

In addition to the ANOVA analyses, we performed linear-mixed effect models to confirm the main effects and interactions between factors (i.e., category base rate, contrast levels, group). For Experiment 1, the model performed on the sensitivity (*d’*) investigated the main effects and interactions with category B base rate (75%, 50%, 25%), contrast levels (0.004, 0.016, 0.033, 0.093, 0.18, 0.36, 0.72), and group (autistic, non-autistic) as fixed factors. The model performed on the decision criterion (*Δ*_criterion_), investigated the main effects and interactions with contrast level (0.004, 0.016, 0.033, 0.093, 0.18, 0.36, 0.72) and group (autistic, non-autistic) as fixed factors. The model performed on the deviation from optimality (*c*_error_) investigated the main effects and interactions with category B base rate (75%, 25%), contrast levels (0.004, 0.016, 0.033, 0.093, 0.18, 0.36, 0.72), and group (autistic, non-autistic) as fixed factors. For Experiment 2, the models performed on the estimated uncertainty (*σ*_sens_), decision boundary (*k*), and deviation from optimality (*k*_error_ ) investigated the main effects and interactions with contrast levels (0.004, 0.016, 0.033, 0.093, 0.18, 0.36, 0.72), and group (autistic, non-autistic) as fixed factors. In all models, subject was added as a random factor.

For both experiments, we employed the Pearson correlation coefficient (*r*) to investigate the relationships between individuals’ deviation from an optimal observer (*c*_error_ in Experiment 1 and *k*_error_ in Experiment 2) and the AQ score. Correlations were calculated for both groups across prior blocks and contrast levels.

Participants’ reaction time was investigated with a mixed-design ANOVA with 3 factors: 1) contrast level (0.004, 0.016, 0.033, 0.093, 0.18, 0.36, 0.72), group (non-autistic, autistic) and prior block (high, neutral, and low) on their reaction time averaged across trials.

**Supplementary Results**

**Experiment 1: the effect of prior knowledge on decision boundary**

**Perceptual sensitivity**

The ANOVA analysis of the effects of base rate block, contrast and group on *d’* revealed a main effect of base rate, *F*(2, 150) = 3.60, *p* = .030, *η_p_²* =.05, with greater sensitivity in the 50% base rate compared to the 25% base rate condition, *t*(538) = 3.17, *p* = .005). We also observed a significant interaction between base rate and contrast level (*F*(12, 900) = 5.95, *p <* .001, *η_p_²* = .07).

Further analyses showed that the interaction is explained by a significant main effect of the base-rate in the contrast level 0.004 (*F*(2, 152) = 3.50, *p* = .033, *η_p_²* = .04), 0.18 (*F*(2, 152) = 7.83, *p* < .001, *η_p_²* = .09), 0.36 (*F*(2, 152) = 7.12, *p* = .001, *η_p_²* = .09), and 0.72 (*F*(2, 152) = 14.53, *p* < .001, *η_p_²* = .16), but not in the contrast level 0.016 (*F*(2, 152) = 2.59, *p* = .078, *η_p_²* = .03), 0.033 (*F*(2, 152) = 0.85, *p* = .429, *η_p_²* < .01) and 0.093 (*F*(2, 152) = 1.71, *p* = 0.185, *η_p_²* = .02). In contrast level 0.004, the main effect of base-rate came from a significantly higher sensitivity in the base rate condition 25% compared to the condition 50% base (*t*(151) = 2.77, *p* = .019). In contrast level 0.18, the main effect of base rate was explained by a higher sensitivity in the base rate condition 50% compared to the condition 75% (*t*(151) = 3.02, *p* = .009). In the contrast level 0.36, the main effect of base-rate was explained by a lower sensitivity in the base rate condition 25% compared to the condition 75% (*t*(143) = 2.61, *p* = .03) and condition 50% (*t*(150) = 2.83, *p* = .016). Finally, in contrast level 0.72, the main effect of base rate was due to a significantly higher sensitivity in the base-rate condition 50% compared to the condition 75% (*t*(152) = 4.53, *p* < .001), and significantly higher sensitivity in the base-rate condition 50% compared to the condition 25% (*t*(150) = 3.09, *p* = .002).

The linear mixed-effect model revealed that only the effect of contrast level on sensitivity was significant, $\beta$ = 0.997, *se* = 0.231, *t*(1534) = 4.219, *p* < .001. Here, the main effect of group ($\beta$ = -0.134, *se* = 0.128, *t*(799) = -1.049, *p* = .295) and the interaction between group and contrast level ($\beta$ = 0.153, *se* = 0.365, *t*(1534) = 0.418, *p* = .676) were not significant. The results of the model are detailed in **Supplementary Results**, **Table 1**.

|  | Estimate | Standard Error | df | Statistic | p-value |
| --- | --- | --- | --- | --- | --- |
| Base rate | 0.101 | 0.135 | 1534 | 0.747 | .455 |
| Contrast | 0.977 | 0.231 | 1534 | 4.219 | <.001 |
| Group | -0.134 | 0.128 | 779 | -1.049 | .295 |
| Base rate X Contrast | -0.183 | 0.429 | 1534 | -0.428 | .670 |
| Base rate X Group | -0.047 | 0.212 | 1534 | -0.220 | .826 |
| Contrast X Group | 0.153 | 0.365 | 1534 | 0.418 | .676 |
| Contrast X Group X Base rate | 0.292 | 0.675 | 1534 | 0.433 | .665 |

**Table 1.** Results of the linear mixed-effect model investigating the main effects and interactions of category base rate, contrast level, and group on the sensitivity (*d’*).

**Decision boundaries**

The linear mixed-effect model were consistent with the ANOVAs results, and revealed a significant main effect of contrast level ($\beta$ = -0.881, *se* = 0.154, *t*(460) = -5.726, *p* < .001), and no significant effect of group ($\beta$ = 0.182, *se* = 0.135, *t*(99) = 1.353, *p* = .179) or interaction between group and contrast level ($\beta$ = 0.284, *se* = 0.243, *t*(460) = -1.169, *p* = .243).

**Suboptimality**

The linear mixed-effect model performed on the suboptimality supported the ANOVA results, by showing a significant main effect of contrast level ($\beta$ =-1.834, *se* = 0.786, *t*(969) = -2.334, *p* = .020), and no main effect of group ($\beta$ = -0.266, *se* = 0.426, *t*(969) = -0.624, *p* = .533) or interaction between group and contrast level ($\beta$ = 0.002, *se* = 1.234, *t*(969) = 0.001, *p* = .999). All the results from the model are detailed in **Supplementary Results**, **Table 2**.

|  | Estimate | Standard Error | df | Statistic | p-value |
| --- | --- | --- | --- | --- | --- |
| Base rate | -0.279 | 0.441 | 969 | -0.631 | .528 |
| Contrast | -1.834 | 0.786 | 969 | -2.334 | .020 |
| Group | -0.266 | 0.426 | 969 | -0.624 | .533 |
| Base rate X Contrast | -0.165 | 1.406 | 969 | -0.117 | .907 |
| Base rate X Group | -0.553 | 0.698 | 969 | 0.793 | .428 |
| Contrast X Group | 0.002 | 1.234 | 969 | 0.001 | .999 |
| Contrast X Group X Base rate | -0.516 | 2.223 | 969 | -0.232 | .817 |

**Table 2.** Results of the linear mixed-effect model investigating the main effects and interactions of category base rate, contrast level, and group on the deviation from optimality (*c*_error_).

**Correlation between AQ and deviation from optimality**

The correlation testing the relation between the AQ and *c*_error_ showed no significant relation between the two variables for either the autistic (*r*(21) = -0.20, *p* = .36) or the non-autistic (*r*(38) = -0.03, *p* = .87) group, as shown by the regression lines in **Supplementary** **Fig. 2a**. These results indicate that the level of autistic traits is not related to the way individuals integrate prior information while making perceptual decisions.


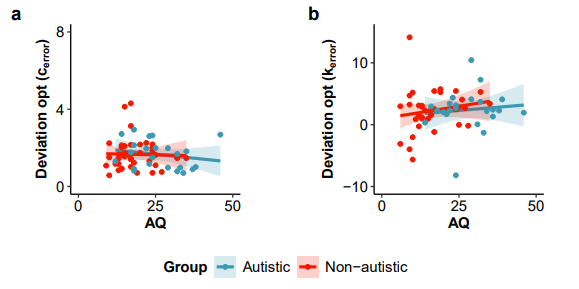


**Supplementary Figure 2.** **Correlation between the deviation from optimality (*c*_error_) and the Autistic Quotient (AQ)**. **(a)** Experiment 1, manipulating the prior. **(b)** Experiment 2, manipulating the sensory uncertainty. The data points represent individuals’ suboptimality across contrast and block. The solid lines represent the linear regression line per group. The sample size constituted 23 autistic and 40 non-autistic participants in **(a)** and 24 autistic and 37 non-autistic participants in **(b)**.

**Reaction time**

The ANOVA analyzing the effects of base rate block, group and contrast level on the averaged reaction time across trials revealed a main effect of group, *F*(1, 73) = 11.47, *p* = .001, *η_p_²* = 0.14, with a significantly higher reaction time in the autistic compared to the non-autistic group, *t*(943) = 8.90, *p* < .001 (**Supplementary** **Figs. 3a-b**). The main effect of contrast level (*F*(6, 438) = 1.08, *p* = .374, *η_p_²* = .02) and base rate block (*F*(2, 146) = 1.80, *p* = .170, *η_p_²* = .02) were not significant. However, the interaction between contrast level and group was significant (*F*(6, 438) = 2.35, p = .030, *η_p_²* = .03), and stemmed from a significantly higher reaction time in the autistic group in every contrast level except the level 0.004. The interaction between group and block (*F*(2, 146) = 1.92, *p* = .150, *η_p_²* = .03), contrast level and base rate block (*F*(12, 876) = 1.71, *p* = .300, *η_p_²* = .02), and the triple interaction between group, contrast level and base rate block (*F*(12, 876) = 0.72, *p* = .730, *η_p_²*  = .01) were not significant. The results are aligned with previous findings showing that autistic participants respond more slowly in a perceptual decision task than non-autistic participants^38^. Furthermore, the nonsignificant effect of contrast indicates that there was no tradeoff between speed and accuracy in both groups.

**
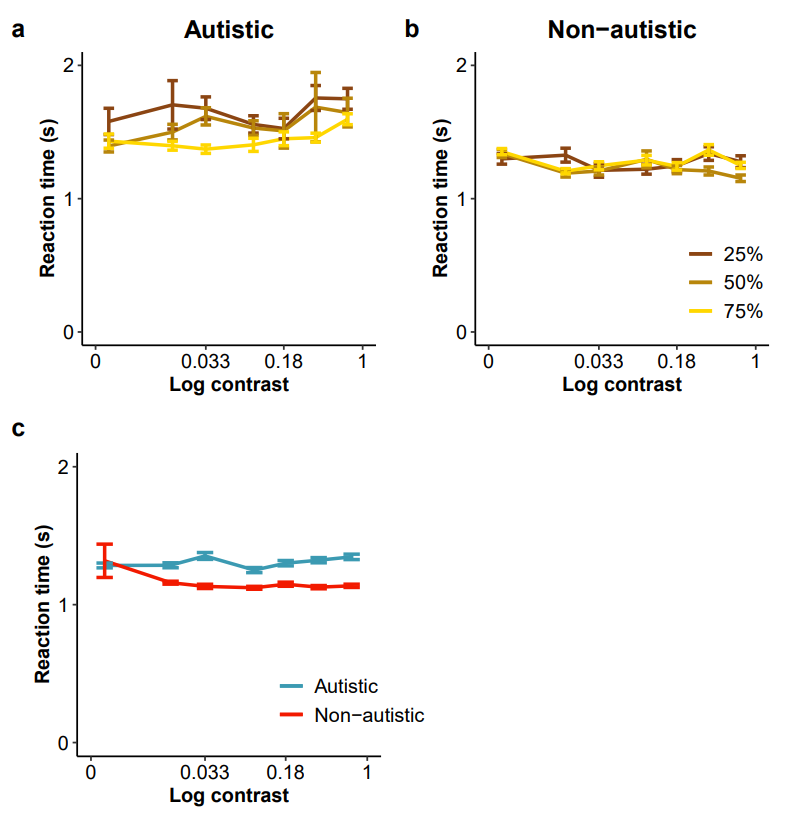
**

**Supplementary Figure 3. Mean reaction time per group and contrast level** for the experiments manipulating **(a, b)** base rate (Exp 1) and **(c)** sensory evidence (Exp 2). The base rate legend represents the probability for Category B to appear. Data points show means across participants and error bars represent ±SE. The sample size constituted 30 autistic and 45 non-autistic participants in the prior experiment **(a, b)** and 24 autistic and 39 non-autistic participants in the sensory uncertainty experiment **(e)**.

**Experiment 2: The effect of sensory uncertainty on decision boundary**

**Perceptual sensitivity**

The linear mixed-effect analyses performed on the sensitivity aligned with the ANOVA results, by showing a main effect of contrast, $\beta$ = -19.573, *se* = 3.663, *t*(400) = -5.343, *p* < .001, and no main effect of group ($\beta$ = -0.208, *se* = 1.938, *t*(150) = -0.107, *p* = .915) or interaction between contrast and group ($\beta$ = -0.983, *se* = 5.770, *t*(400) = -0.170, *p* = .865).

**Decision boundaries**

The linear mixed-effect analyses supported the findings from the ANOVA by showing a main effect of contrast ($\beta$ = -14.978, *se* = 2.835, *t*(400) = -5.284, *p* < .001), and no main effect of group ($\beta$ = -0.277, *se* = 1.570, *t*(138) = -0.176, *p* = .860) or interaction between group and contrast ($\beta$ = 0.228, *se* = 4.465, *t*(400) = 0.051, *p* = .959).

**Suboptimality**

Although the main effect of contrast was not significant in the linear mixed-effect model ($\beta$ = -1.691, *se* = 1.085, *t*(388) = -1.558, *p* = .120), there was no main effect of group ($\beta$ = 0.760, *se* = 0.887, *t*(86) = 0.857, *p* = .394), or interaction between contrast and group ($\beta$ = -2.136, *se* =1.684, *t*(388) = -1.268, *p* = .205), supporting the previous findings suggesting no difference in deviation from an optimal observer between groups.

**Correlation between AQ and deviation from optimality**

The analysis of the correlation between AQ and *c*_error_ revealed no significant relation between the two variables for the autistic (*r*(22) = 0.11, *p* = .62) and the non-autistic (*r*(35) = 0.12, *p* = .48) groups (**Supplementary** **Fig. 2b**). These results, indicating that in both groups, the deviation from an optimal observer is not mediated by autistic traits, supported the findings that autistic individuals integrate the sensory uncertainty information in a typical manner.

**Reaction time**

The mixed-design ANOVA investigating the effect of group and contrast level on the reaction time revealed a main effect group (*F*(1, 61) = 6.74, *p* = .012, *η_p_²* = .10) with a significantly higher reaction time in the autistic compared to the non-autistic group, *t*(385) = 4.72, *p* < .001 (**Supplementary** **Fig. 2c**). The effect of contrast (*F*(6, 366) = 1.00, *p* = .428, *η_p_²* = .02), and the interaction between group and contrast *F*(6, 366) = 1.92, *p* = .076, *η_p_²* = .03 were not significant. Once again, we replicated the higher reaction time for the autistic group, and as in Experiment 1, there was no tradeoff between speed and accuracy in Experiment 2.
